# Structural Requirements for *Yersinia* YopJ Inhibition of MAP Kinase Pathways

**DOI:** 10.1371/journal.pone.0001375

**Published:** 2008-01-02

**Authors:** Yi-Heng Hao, Yong Wang, Dara Burdette, Sohini Mukherjee, Gladys Keitany, Elizabeth Goldsmith, Kim Orth

**Affiliations:** 1 Department of Molecular Biology, University of Texas Southwestern Medical Center at Dallas, Dallas, Texas, United States of America; 2 Department of Biochemistry, University of Texas Southwestern Medical Center at Dallas, Dallas, Texas, United States of America; 3 School of Life Science, Arizona State University, Tempe, Arizona, United States of America; 4 University of Washington School of Public Health, Seattle, Washington, United States of America; University of Queensland, Australia

## Abstract

MAPK signaling cascades are evolutionally conserved. The bacterial effector, YopJ, uses the unique activity of Ser/Thr acetylation to inhibit the activation of the MAPK kinase (MKK) and prevent activation by phosphorylation. YopJ is also able to block yeast MAPK signaling pathways using this mechanism. Based on these observations, we performed a genetic screen to isolate mutants in the yeast MKK, Pbs2, that suppress YopJ inhibition. One suppressor contains a mutation in a conserved tyrosine residue and bypasses YopJ inhibition by increasing the basal activity of Pbs2. Mutations on the hydrophobic face of the conserved G α-helix in the kinase domain prevent both binding and acetylation by YopJ. Corresponding mutants in human MKKs showed that they are conserved not only structurally, but also functionally. These studies reveal a conserved binding site found on the superfamily of MAPK kinases while providing insight into the molecular interactions required for YopJ inhibition.

## Introduction

The bacterial pathogen *Yersinia pestis* was the causal agent of three pandemics referred to as the Plague of Justinian, Black Death and the Third Pandemic [1]. Two other related bacterial pathogens, *Yersinia pseudotuberculosis and Yersinia enterocolitica* are causal agents for gastroenteritis [1]. All of the three *Yersinia* species harbor a 70 Kb virulence plasmid that encodes a Type III Secretion System (TTSS) and effector proteins [2]. The TTSS functions like a syringe, whereby it injects or translocates effectors, also referred to as Yops (Yersinia outer proteins), from the bacterial cell into the infected host cell [2]. Yops are essentially bacterial equivalents of viral oncogenes in that they mimic or capture a eukaryotic activity to target and manipulate eukaryotic signaling machineries during infection. However, unlike viral proteins that are made in the host, effectors are made in bacteria and therefore must be kept in a quiescent state until after they are translocated into the host. The effectors are silenced in bacteria by a number of mechanisms including association with a chaperone and/or lack of a eukaryotic activator or substrate [3].

One of the *Yersinia* effectors, YopJ, is a 32 kD effector protein that disrupts the innate immune response and promotes apoptosis in infected cells by blocking the MAPK pathways and the NFκB pathway [1], [4]. YopJ disrupts these signaling systems by blocking the activation of all MAP kinase kinases (MKKs) and IKKβ (but not IKKα)[4]. YopJ requires an intact catalytic triad consisting of the concserved residues His, Cys and Glu/Asp for its inhibitory activity [5]. Previous over-expression studies implicated various mechanisms for YopJ's inhibitory activity, but failed to identify a specific substrate [5]–[8]. Recently it was discovered that YopJ is an acetyltransferase that inhibits the activation of these kinases by modifying serine and/or threonine residues in the activation loop thereby preventing their modification by phosphorylation [9], [10]. It is predicted that YopJ uses a two-substrate, ping-pong mechanism similar to that used by a cysteine protease, whereby the first substrate interacts with the enzyme, forming a high-energy acyl-enzyme intermediate, that is attacked by the second substrate resulting in a modified product [10], [11]. However, in contrast to a protease that uses a peptide and water for substrates, YopJ uses acetyl-CoA and a hydroxyl from the R group on an amino acid.

The mechanism YopJ uses to inhibit the MAPK pathways is evolutionarily conserved because YopJ was able to inhibit the MAPK pathways in *Saccharomyces cerevisiae*, including the mating and HOG pathways [12]. Herein, we established that YopJ blocks the HOG pathway by blocking activation of the yeast MKK, Pbs2, by acetylation. Based on these observations, we performed a targeted suppressor screen in yeast using YopJ's inhibitory effect on HOG MAPK pathway to help gain further insight into the molecular mechanism of YopJ inhibition of the MKKs and the interactions between MKKs and YopJ.

A genetic screen was performed to isolate suppressor mutations in Pbs2 that would allow growth on hyper-osmotic media in the presence of YopJ. We identified a novel mutation in conserved tyrosine that when mutated results in a constitutively active kinase. Other suppressor mutations in Pbs2 revealed a conserved binding site for YopJ on the MKKs. These studies not only provide mechanistic insight into the inhibitory activity of the *Yersinia* effector YopJ but also support predictions for mechanisms involved in the activation of the kinases that are sensitive to YopJ's inhibition.

## Results

### YopJ binds, acetylates and inhibits activation of Pbs2

**Figure 1:**
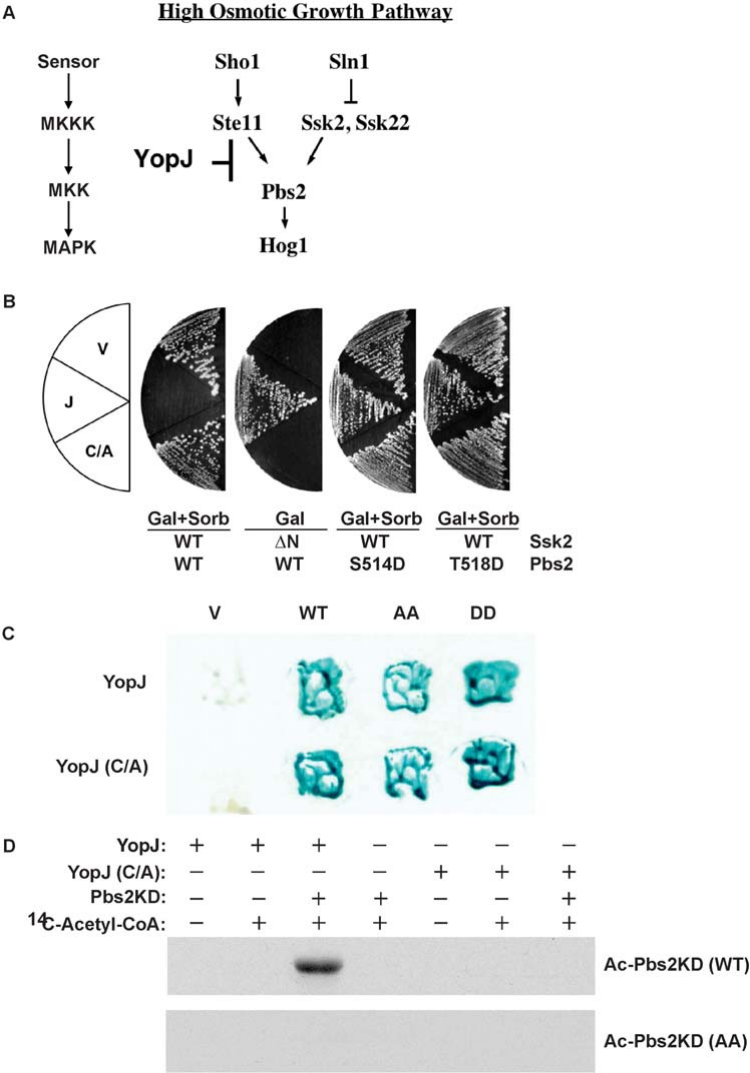
YopJ binds, acetylates and inhibits activation of Pbs2. (A) Schematic representation of the canonical MAPK pathway aligned with the yeast HOG MAP kinase pathway. (B) Yeast *pbs2Δ* strain is cotransformed with pRS416-GPD1-Pbs2 wild type, S514D or T518D mutants and pRS413-Gal1 vector, YopJ or YopJ(C172A) mutant. pRS415-Gal1-Ssk2ΔN mutant is also transformed to the pRS416-GPD1-Pbs2 wild type strains (the third panel). Strains are plated on media containing 2% glucose or 2% raffinose, 1% galactose with or without 1 M sorbitol and incubated at 30°C. (C) Yeast two-hybrid assay of the interaction between YopJ or YopJ(C172A) mutant and Pbs2 wild type, Pbs2 dead mutant (AA) or Pbs2 constitutively active mutant (DD). (D) Recombinant YopJ or YopJ(C172A) are incubated with or without purified Pbs2 kinase domain (Pbs2KD(WT)) or Pbs2 (S514A, T518A) mutant (AA) kinase domain (Pbs2KD(AA)) in the presence or absence of ^14^C-labeled acetyl-CoA at 30°C for 1 hour. Samples are separated by SDS-PAGE and analyzed by autoradiography.

We demonstrated that YopJ is able to block the HOG MAPK signaling pathway downstream of the negative regulator Sln1 (Figure 1A) [12]. As shown in Figure 1B, the growth of yeast expressing YopJ is arrested when grown on galactose/sorbitol plates, while strains containing either the empty vector or expressing the catalytically inactive YopJ-C172A mutant (YopJ-C/A) are able to grow on galactose/sorbitol selective media. To confirm that YopJ blocks the HOG pathway in a manner similar to that observed for the mammalian MAPK pathways by preventing activation of MKKs, we generated a yeast strain that contained an activated form of yeast MKKK Ssk2 (Ssk2ΔΝ) under the control of a galactose inducible promoter (Figure 1B) [13]. We observed that growth of the Ssk2ΔΝ strains on galactose was arrested due to constitutive activation of the HOG pathway in the absence of a hyper-osmotic growth environment (Figure 1B) [13]. However, co-expression of YopJ in this cell line rescued the growth arrest phenotype, thereby supporting the hypothesis that YopJ inhibits the HOG pathway downstream of the MKKK (Figure 1A, B).

To further confirm whether YopJ inhibits the signaling pathway by preventing activation of the Pbs2 (yeast MKK) in the HOG pathway, phospho-mimic mutants of Pbs2, in which either the serine or threonine in the activation loop of the kinase were replaced with aspartate (Pbs2(S514D) or Pbs2(T518D)), were coexpressed with an empty vector, YopJ or YopJ-C/A mutant. We observed that both mutants grow in the presence of 1M sorbitol and YopJ (Figure 1B). These observations support the findings of previous cell epistasis experiments, whereby YopJ was unable to block the mammalian ERK MAPK signaling pathway downstream of the activated MKK1-EE [4].

We next assessed whether YopJ could bind to the yeast MKK Pbs2, using yeast two-hybrid analysis. As previously observed for mammalian MKK [4], both YopJ and YopJ-C172A were able to interact with wild type Pbs2 (Figure 1C). Two hybrid analysis with a panel of Pbs2 mutants, including inactive Pbs2 (Pbs2(S514A;T518A)), and activated Pbs2 (Pbs2(S514D;T518D)), revealed that changes in amino acids that cause a change in the charge of the activation loop and/or the activity of the enzyme do not affect the binding of YopJ to Pbs2 (Figure 1C).

Recently, it was demonstrated that YopJ prevents the activation of MKKs by acetylating the serine and threonine residues in the MKK activation loop, thereby preventing their phosphorylation by upstream kinases [9], [10]. We confirmed that the Pbs2 kinase domain (331–668 aa) is a substrate for YopJ acetylation (Figure 1D). The substrate specificity of YopJ for the SXXXT motif in the activation loop of Pbs2 was confirmed using site-directed mutagenesis of this motif. A single mutation in Pbs2 of either the Ser514 or Thr518 to an alanine results in a substrate that can be acetylated by YopJ (data not shown). However, the double mutation in Pbs2 (Ser514Ala;Thr518Ala) produced a substrate that is no longer acetylated by YopJ, supporting the proposal that these are the two residues that are modified and preventing phosphorylation (Figure 1D).

### Isolation of suppressor mutations in Pbs2

YopJ inhibits growth of yeast in the presence of sorbitol (Figure 1B, 2A) [12]. We screened a PCR random mutagenesis library of Pbs2 (∼one mutation/kb DNA) for suppressor mutations in Pbs2 that would allow cells to grow on media with 1M sorbitol when YopJ was expressed. The screen was carried out in the *pbs2Δ* haploid strain to ensure that the suppressor mutations in Pbs2 contained the following two properties: (1) the mutants are enzymatically active, and (2) the mutants suppress growth inhibition induced by YopJ expression.

**Figure 2:**
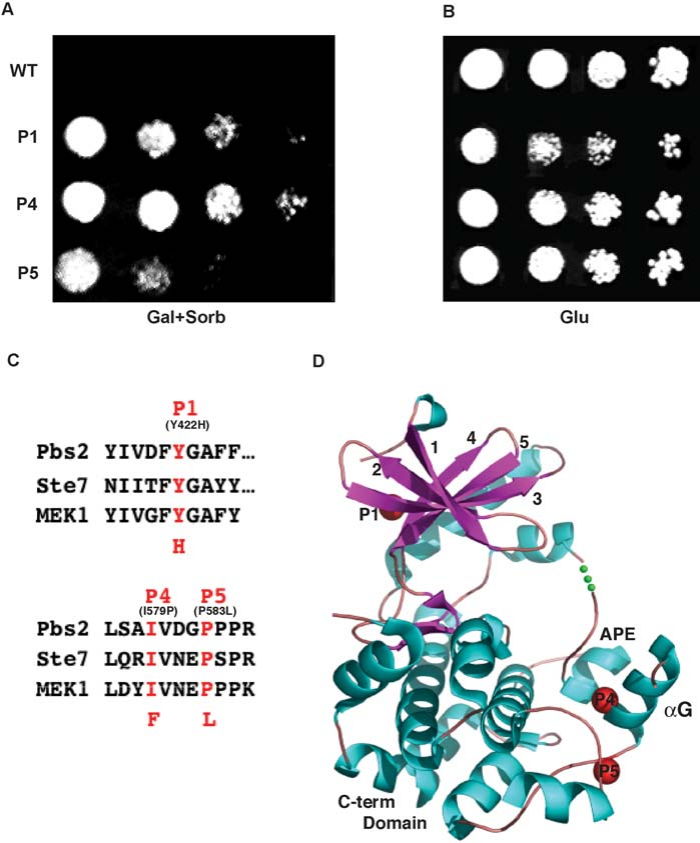
Pbs2 mutants that suppress YopJ inhibition thus survive on sorbitol plate. (A) Dilutions of yeast BY4741-*pbs2Δ* strain co-transformed with YopJ and Pbs2 wild type or mutants are plated on synthetic media containing 2% raffinose, 1% galactose and 1 M sorbitol and incubated at 30°C. (B) Dilutions of yeast BY4741-*pbs2Δ* strain cotransformed with YopJ and Pbs2 wild type or mutants are plated on synthetic media containing glucose and incubated at 30°C. (C) Sequence alignment shows all screened mutants are conserved in MKKs from yeast to human. (D) Structural mapping of the screened mutants on a canonical MKK structure: P1 on beta strand 4; P4 on the G α-helix; P5 at the end of the G α-helix.

We recovered eighty strains containing suppressor mutations in Pbs2 from the 9×10^4^ transformants screened. Pbs2 is a scaffolding kinase with domains that bind both the upstream MKKK and the downstream MAPK Hog1 [14]. We narrowed our focus to mutations found exclusively in the kinase domain of Pbs2 and to residues that are conserved in the mammalian and yeast MKKs. After elimination of number of mutants, we finally focused on three unique suppressor mutants (P1, P4, P5) that prevented YopJ inhibition of the HOG MAPK signaling pathway as indicated by growth in the presence of sorbitol (Figure 2A).

### Suppressor mutations in Pbs2 map to evolutionarily conserved residues

The three suppressor mutations in Pbs2 are due to changes in amino acids that are evolutionarily conserved in all the MKK family members (Figure 2C). The mutations map to three locations on a canonical MKK structure: P1, on the center of beta strand 4 in the conserved motif X-F-Y-G-A-X; P4 and P5, on and at the end of the G helix, respectively (Figure 2D)[15]. These findings are consistent with our previous observation that YopJ interacts with and inhibits MKK family members by a common mechanism [10], [12].

We predicted that the suppressor mutations in Pbs2 could inhibit YopJ by a variety of mechanisms. If the suppressor mutants had increased basal activity, they would bypass YopJ inhibition in a manner similar to that observed for Pbs2(S514D) and Pbs2(T518D) (Figure 1B). Therefore, we tested whether any of the suppressor mutations in Pbs2 contained increased basal activity or constitutive activity by plating the yeast strains on glucose in the absence of osmotic stimulus. Out of the three suppressor mutants, P1, grew more slowly on glucose media when compared to the other yeast strains grown on plates or in liquid media containing glucose as a carbon source (Figure 2B and data not shown, respectively), supporting the hypothesis that this mutant might bypass the growth inhibition induced by YopJ with increased basal kinase activity.

**Figure 3:**
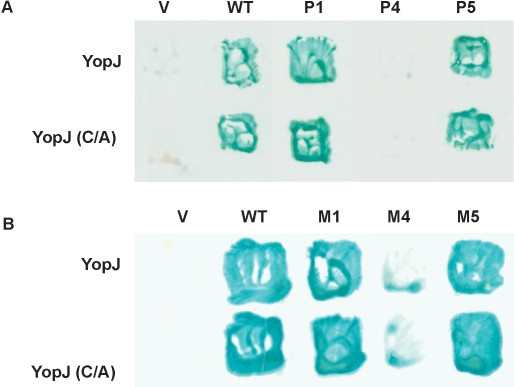
Interaction between YopJ and Pbs2 or MKK1 mutants. (A) Yeast two-hybrid assay of the interaction between YopJ or YopJ(C172A) mutant and Pbs2 wild type or Pbs2 mutants from the screening. (B) Yeast two-hybrid assay of the interaction between YopJ or YopJ(C172A) mutant and corresponding MKK1 mutants.

From previous studies we have observed that YopJ inhibits only the kinases to which it binds (all MKKs and IKKβ) but not kinases it does not bind (IKKα, Raf, p38, ERK1/2, Jnk) [4]. Therefore, we predicted that one mechanism to suppress YopJ inhibition would be to prevent binding of YopJ to its target kinase. Using the yeast two-hybrid assay, we analyzed whether the suppressor mutants could bind to YopJ and observed that one of these mutants, P4, was unable to interact with YopJ (Figure 3A). Because the remaining suppressor mutants (P1, P5) were able to bind to YopJ, we propose that binding of YopJ to Pbs2 may be necessary but not sufficient for YopJ's inhibition. As expected, analogous mutations made in mammalian MKK1, resulted a similar profile of binding to YopJ as assessed by yeast two-hybrid (Figure 3B), reinforcing the proposal that YopJ inhibits the MKKs by a common mechanism [4], [10].

**Figure 4:**
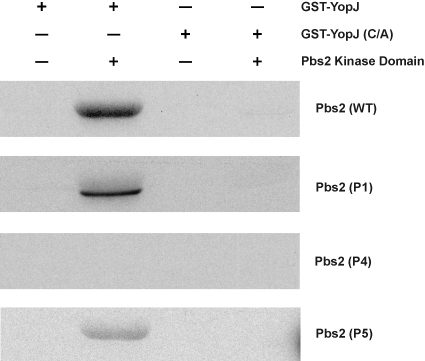
Acetylation by YopJ of Pbs2 mutants is impaired. Recombinant YopJ or YopJ(C172A) is incubated with purified Pbs2 kinase domain or its mutants in the presence and absence of ^14^C-labeled acetyl-CoA for 1 hour at 30°C. Samples are separated by SDS-PAGE and analyzed by autoradiography.

Another possible mechanism for suppression of YopJ inhibition would be that YopJ cannot acetylate the mutants. To test this hypothesis, the kinase domain from each of the suppressor mutants was purified from bacteria and tested as a substrate for YopJ in an *in vitro* acetylation reaction. We observed that the kinase domains of both wild type and P1 kinase domains were efficiently acetylated, but not that of P5 (Figure 4). As predicted, the P4 mutant, which does not bind to YopJ, cannot be acetylated (Figure 4).

Finally, we tested whether the mutants could suppress the YopJ-mediated inhibition of growth, when the mutants are co-expressed with Pbs2. We observed that P1, but not P4 or P5, co-expressed with the endogenous Pbs2 could grow in the presence of YopJ (see Figure S2).

Overall, the isolated mutants show three distinct phenotypes: 1. increased basal activity and suppression of YopJ in the presence of endogenous Pbs2 (P1); 2. inability to bind and be acetylated by YopJ, but unable to suppress YopJ in the presence of endogenous Pbs2 (P4); and 3. inefficient acetylation by YopJ and unable to suppress YopJ in the presence of endogenous Pbs2 (P5). Below, we delineate the properties and mechanisms utilized by each suppressor mutant to prevent YopJ inhibition of MAPK signaling.

### P1 exhibits an increase in basal kinase activity

**Figure 5:**
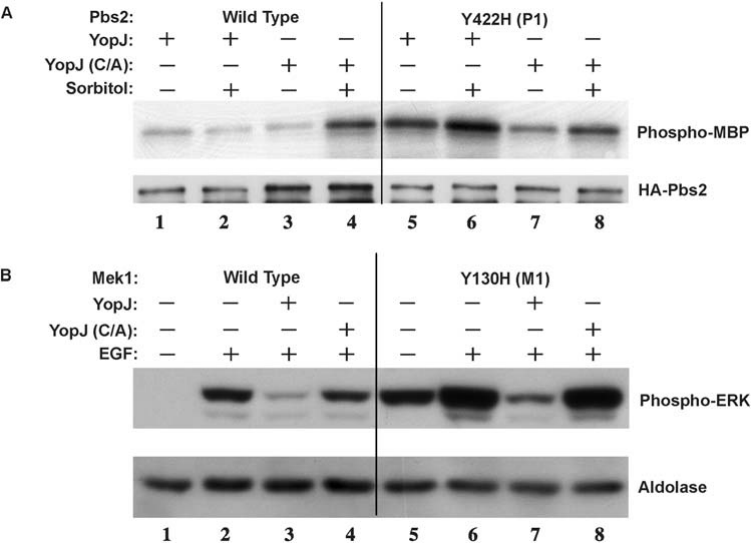
P1 mutant exhibits a higher basal kinase activity. (A) Pbs2 wild type or P1 mutants expressed in yeast *pbs2Δ* strain with or without sorbitol stimulation are purified and incubated with MBP and ^32^P-γ-ATP at 30°C for 30 minutes. The samples are separated by SDS-PAGE, transferred to PVDF membrane and then analyzed by autoradiography. The same membrane is probed with an anti-HA antibody. (B) HEK293 cells are transfected with either Flag-tagged YopJ or YopJ(C172A), HA-tagged ERK and HA-tagged MKK1 wild type or M1 mutant. Cells are lysed and immunoprecipitated using an anti-HA antibody and Protein G beads. Beads are separated using SDS-PAGE and probed with either anti-HA or anti-phospho ERK antibodies.

Based on growth assays on glucose, the P1 mutant would be expected to exhibit a higher basal kinase activity than the wild type Pbs2. To test this hypothesis, we immunopurified wild type and mutant forms of Pbs2 from yeast that were exposed to normal and hyperosmotic conditions and analyzed them in an *in vitro* kinase assay. As seen in Figure 5A, the P1 mutant protein, exhibits high basal kinase activity in the absence of stimulus when compared to wild-type Pbs2. As the tyrosine residue that is mutated in P1 (Y423) is conserved in all MKKs, we tested whether the same mutation in mammalian MKK1 (Y130) would alter its basal activity. Mutation of the conserved tyrosine to a histidine in the mammalian MKK1 also resulted in high basal activity (Figure 5B). As expected, MKK1 kinase activity is diminished in the presence of YopJ, because the endogenous kinases are inhibited leaving only the mutant kinases active (Figure 5B). These results support our previous findings that YopJ inhibits the activation of MKKs and that acetylation by YopJ cannot inhibit kinases that are constitutively active (Figure 1) In addition, the P1 mutant might reveal a novel mechanism to activate a kinase.

### P4 and P5 mutants reveal the binding site for YopJ

**Figure 6:**
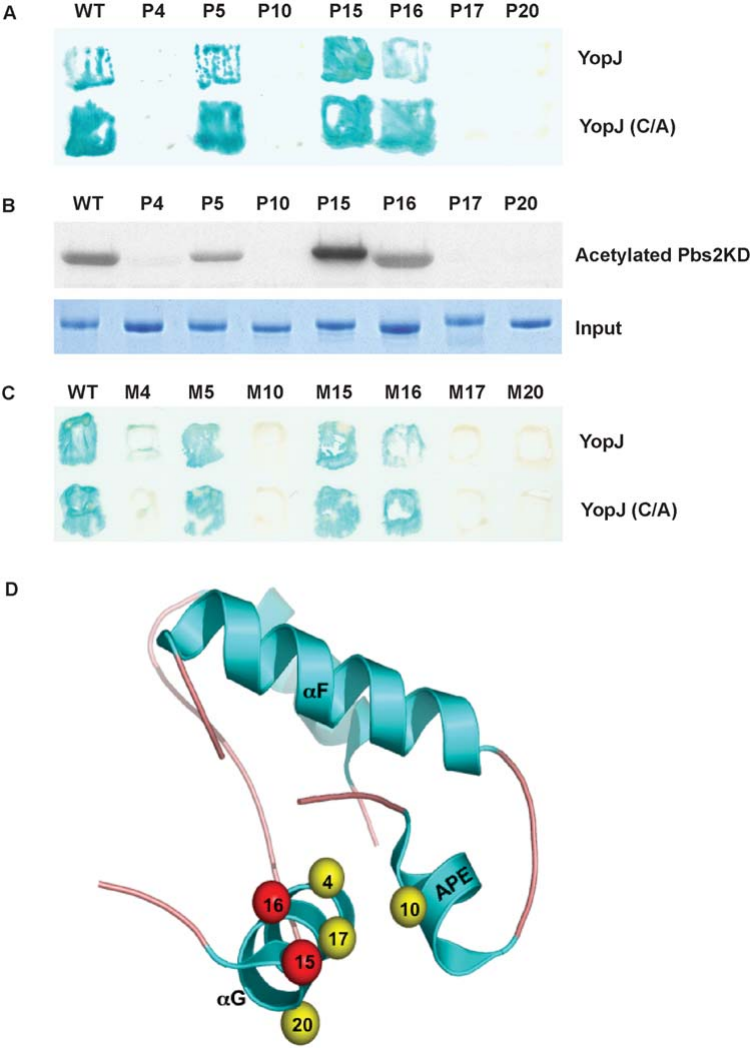
The binding site on MKKs for YopJ. (A) Yeast two-hybrid assay of the interaction between YopJ or the YopJ(C172A) mutant and wild type Pbs2 or mutants of Pbs2 in or around G α-helix. (B) Recombinant YopJ is incubated with purified Pbs2 kinase domain or mutants in or around the G α-helix in the presence of ^14^C-labeled acetyl-CoA for 1 hour at 30°C. Samples are separated by SDS-PAGE and analyzed by autoradiography. Substrate loading was controlled by protein staining. (C) Yeast two-hybrid assay of the interaction between YopJ or YopJ (C172A) mutant and corresponding MKK1 mutants. (D) Structural mapping of G α-helix and APE domain, showing the mutants tested above.

The P4 mutation is located in Subdomain X on the small G α-helix at the base of the carboxyl terminal lobe of the kinase domain (Figure 2D). The mutation of a conserved isoleucine to a phenylalanine disrupted the binding of YopJ to the mutant kinase (Figure 3A). The P4 mutation is located on the side of the α-helix that is exposed to solvent and we reasoned that this region might be the binding site on the kinase domain for YopJ (Figure 6D). To further investigate this possibility, we made a series of alanine mutations (P15, P16, P17, and P20) in the conserved residues along the G α-helix (Figure 6D). All of these mutants produced functional kinases and were able to rescue the growth defect of *pbs2Δ* strain on hyper-osmotic media (Figure S1B). Mutants P15 and P16, but not mutants P17 and P20, were able to interact with YopJ, demonstrated by yeast two-hybrid analysis (Figure 6A). We next assessed whether the kinase domain from each of these mutants was a substrate for YopJ in an acetyltransferase assay. Only the mutants that bound to YopJ, P15 and P16, were acetylated by YopJ (Figure 6B). The P17 and P20 mutants on the G α-helix of the kinase domain did not bind to YopJ and were not acetylated (Figure 6B). In contrast to P15 and P16 mutants, the P4, P17 and P20 mutants are located on the same side of the G α-helix that faces the APE-loop (see below).

Further analysis of the YopJ binding site revealed that the face of the G α-helix that binds YopJ opposes the APE loop in the kinase domain (Figure 6D). All MKKs, from yeast to human and IKKβ have either an isoleucine or a leucine residue facing the binding site on the G α-helix. However, unlike the aforementioned kinases that bind to YopJ, IKKα, which does not bind YopJ based on *in vitro* pull down assays, has a phenylalanine at this position [16]. Therefore, we tested whether a Pbs2 mutant containing Ile531Phe (labeled P10) is an active kinase, binds to YopJ and is acetylated by YopJ. We found that the P10 mutant was an active kinase because it was able to rescue the hyper-osmotic sensitive phenotype of *pbs2Δ* strain (Figure S1B) but P10 is unable to bind to or be acetylated by YopJ (Figure 6A and 6B).

The last mutation identified from this genetic screen, P5, is located at the end of the G α-helix and contains a leucine instead of a proline. Although YopJ can bind to P5, we observed that YopJ inefficiently acetylated P5 when compared to the wild type Pbs2 kinase domain (Figure 4 and 6B).

Further analysis of this binding site reveals that the YopJ binding site is conserved in other MKKs. Two-hybrid analysis with similar mutants in MKK (M4, M5, M15, M16, M17, and M20) demonstrates that YopJ cannot bind to the predicted interface of the helix if the corresponding residues are mutated or a phenylalanine is present on the APE loop (Figure 6C).

## Discussion

MAPK pathways are conserved from yeast to man and each of the corresponding kinases in the cascade are activated by a similar mechanism. *Yersinia* strains express an effector protein, YopJ, that inhibits the activation of the second kinase, MKK, in these signaling pathways by acetylating residues that are normally phosphorylated by the upstream kinase. From our genetic screen, we discovered that a mutation in an evolutionarily conserved tyrosine residue is sufficient to increase the basal activity of MKKs. We found that the G α-helix is the binding site on MKKs for YopJ. If YopJ is unable to interact with this site, it cannot acetylate its substrate, MKK.

### P1 mutation of the evolutionarily conserved tyrosine residue in MKK is linked to Cardio-facial-cutaneous Syndrome

In the set of the evolutionarily conserved mutations, we isolated the P1 mutant, in which a conserved tyrosine residue is mutated to a histidine residue. The resulting suppressor mutant displays increased basal activity in the absence of stimulus as observed in both *in vitro* kinase assays and growth assays. We observe that mutation of mammalian MKK at this position also results in a kinase with high basal activity. We propose that mutation of the conserved tyrosine residue results in an activated kinase by a mechanism that is independent of phosphorylation. Therefore, although the P1 suppressor mutant can still bind to YopJ and be efficiently acetylated by YopJ, it bypasses the YopJ block and activates the HOG MAPK pathway.

Recently, Rodriguez-Viciana and colleagues reported on the genetics of activating mutations in the mammalian MAPK pathway that cause Cardio-facial-cutaneous syndrome (CFC) [17] (Online Mendelian Inheritance in Man (OMIM 115150)), a disorder associated with cardiac defects, craniofacial features, epidermal defects in hair and skin, and delay in development. One of the activating mutations is found in MKK1 at the conserved tyrosine (Y130) that aligns with the tyrosine residue mutated in P1 (Y423H). Therefore, in two completely different genetic surveys, using two distantly related species, an activating mutation in the same conserved tyrosine residue was isolated. Future structural studies comparing the wild-type inactive kinase with the active mutant kinase, may reveal evolutionarily conserved structural transition states important for the activation of this family of kinases.

### YopJ-binding site is on the G α-helix on MKKs

Using the Pbs2 suppressor screen we discovered that the G α-helix is the binding site on MKKs for YopJ. Mutations along the side of the G α-helix that is opposite of the APE loop disrupt the interaction between YopJ and Pbs2p. Analogous mutations in the G α-helix of mammalian MKK1 also disrupt binding of YopJ, supporting the proposal that YopJ uses this region of the kinase as a binding site on all members of the MKK family. Based on the identification of the binding site for YopJ on MKKs, we have learned at least three characteristics about the mechanism of YopJ inhibition of the family of MKKs. The first and most obvious is that YopJ must bind to its substrate to acetylate and inhibit it.

Second, we gained insight into why YopJ can inhibit some kinases but not others [4]. During the early studies on the mechanism of YopJ inhibition of signaling pathways, it was observed that YopJ could bind all the mammalian MKKs and IKKβ (but not IKKα) and these kinases were known to play integral roles in the induction of cytokine signaling and the innate immune response. Based on our findings herein, we observe that IKKα, but not IKKβ or members of the MKK family, has a bulky phenylalanine residue instead of an isoleucine or leucine residue on its APE loop, which might block YopJ's access to the binding site on this kinase (Figure 6D). Mutation of Pbs2 at this site to a phenylalanine creates a functional and inducible kinase. However, the Pbs2(I531F) mutant acts as a YopJ suppressor, because it no longer binds and, therefore, is not acetylated by YopJ. It is reasonable to propose, from a mechanistic viewpoint, that the reason YopJ cannot bind to IKKα is because the binding site on the kinase is blocked. In contrast to these observations, Mittal and colleagues observed YopJ-mediated acetylation of IKKα and IKKβ in transient transfection studies [9]. Both IKKα and IKKβ are proposed to form heterodimers[18], and therefore, YopJ is able to interact with this complex via IKKβ and possibly acetylating both kinases. Support of this hypothesis comes from the observation that Pbs2(I531F) is a recessive mutant. Co-expression of Pbs2(I531F) with wild type Pbs2 results in a yeast strain that is inhibited for growth on high osmotic media (data not shown). In a manner similar to the IKKα/IKKβ heterodimer, the Pbs2/Pbs2I531F heterodimer may be susceptible to YopJ inhibition.

Finally, the third characteristic involves the orientation of the binding site on the kinase. P5 contains a mutation in a proline residue at the end of G α-helix and the proline is speculated to be a helix breaker. We hypothesize that the P5 suppressor mutant, Pbs2(P584L) contains a G α-helix that is extended by one amino acid and forms a structure that is not optimal for YopJ inhibition because it alters orientation of the YopJ binding site in Pbs2 and, as a result, we observe the P5 suppressor mutant is inefficiently acetylated by YopJ.

### YopJ inhibition and MKK activation

Herein we have analyzed the inhibition of MAPK signaling by *Yersinia* YopJ using yeast genetics. YopJ inhibits the MKK in the HOG MAPK pathway by acetylation, thereby preventing its activation by phosphorylation (Figure 1A). We have identified suppressor mutants in the yeast Pbs2 that prevent YopJ from inhibiting the MAPK pathways. The first mutant, P1, suppresses YopJ inhibition by bypassing the requirement of phosphorylation for activation. The second, P4, alters the binding site on MKKs so that YopJ is unable to bind and modify the kinase. The third, P5, is predicted to alter the orientation of the binding site for YopJ such that it no longer efficiently modifies its substrate. Retrospectively, it was fortunate that the pbs2 deletion strain is viable, because all of the G α-helix mutants would not have been discovered in a screen using the wild type yeast strain. Our findings have led to a further understanding on how YopJ interacts with and modifies its substrates. As Type III effectors are predicted to mimic or usurp a eukaryotic catalytic activity, we believe the YopJ-binding site we identified on the conserved G-helix will be an important binding platform for eukaryotic signaling molecules that interact with this family of kinases.

## Materials and Methods

### Yeast Strains and Plasmids

HA tagged Pbs2 and mutants were generated in the yeast expression vector pRS416-GPD1 and transformed into a *pbs2Δ* strain or its parental strain, BY4741 (Research Genetics), together with YopJ or YopJ(C172A) mutant encoded by a galactose inducing vector pRS413-Gal1, by the lithium acetate procedure [19]. Recombinant proteins were cloned into pET28a (His tagged) or pGEX (GST tagged) vectors. Mutants were generated using the Quickchange site-directed mutagenesis kit (Stratagene).

### Yeast Culture

Yeasts were cultured at 30°C on plates or in liquid media containing 2% glucose or 2% raffinose and 1% galactose in the presence or absence of 1 M sorbitol. Specific amino acids were excluded from the media for selection purposes. Dilution plating experiments were performed using 5 µl cells per spot, starting with an OD_600_ of 0.1 and serially diluting 5 times each step.

### Genetic Screening of Pbs2 Mutants

A Pbs2 random mutagenesis library (9×10^4^ mutants) was generated with a Stratagene kit and cloned into the pRS416-GPD1 vector. The library was transformed into yeast strain BY4741 *pbs2Δ* strain together with pRS413-Gal1-YopJ and selected for growth on plates with 2% raffinose, 1% galactose and 1 M sorbitol. A total 80 positive clones were identified, 24 of which had a single mutation within the Pbs2 kinase domain.

### Recombinant Protein Purification

Constructs of recombinant proteins were transformed into *E. coli* BL21-DE3 strain and proteins were expressed by growing cells in 2×YT media and inducing at an OD_600_ of 0.6∼0.8 with 0.4 mM IPTG. Cells were lysed by a cell disruptor and N-terminal His tagged proteins (Pbs2 kinase domain and mutants, YopJ) were purified using standard Ni^++^-NTA affinity chromatography purification protocols (Qiagen), while N-terminal GST tagged YopJ was purified as indicated before [10]. After concentration and buffer exchange, purified proteins were stored frozen in the presence of 10% glycerol.

### *In vitro* Acetylation Assay

*In vitro* acetylation assays were performed as described [10]. Briefly, recombinant YopJ or YopJ(C172A) was incubated with purified Pbs2 kinase domain or its mutants in the presence and absence of ^14^C-labeled acetyl-CoA for 1 hour at 30°C. Samples were separated by SDS-PAGE and analyzed by autoradiography.

### Immunoprecipitation from Yeast Extract and Kinase Assay

Yeasts *pbs2Δ* strains co-expressing Pbs2 and YopJ were cultured in media containing 2% raffinose and 1% galactose, harvested, stimulated with 0.7 M sorbitol for 2.5 minutes, and lysed with 1% Triton X-100 in 25 mM Tris, pH 7.5 and 150 mM NaCl, supplied with protease and phosphatase inhibitors. HA tagged Pbs2 was pulled down with an anti-HA antibody (Covance) and Protein G Sepharose beads (Roche, Germany), which were washed with the same buffer with 500 mM NaCl to eliminate nonspecific binding. Kinase assays were performed as previously described [20] using MBP as substrate. Briefly, purified Pbs2 on HA beads were incubated with MBP in the presence of ^32^P-γ-ATP, DTT and cold ATP at 30°C for 30 minutes, separated by SDS-PAGE, and then analyzed by autoradiography.

### Mammalian MKK1 Phosphorylation Experiment

HEK293 cells plated at a density of 0.5×10^6^ cells/ml were transfected with either flag-tagged YopJ or YopJ C/A, HA-tagged ERK and various HA-tagged MKK1 mutants. Cells were lysed in HNT buffer containing protease inhibitors (0.01 M Hepes, pH 7.4, 0.05 M NaCl, 1% Triton X-100, 0.5 mM Na_3_VO_4_, 0.5 mM EGTA, 0.02 M NaF, 0.02 M β-glycerophosphate, and 1 mM DTT) and the HA-MKK was immunoprecipitated using an anti-HA antibody (Covance) and Protein G Sepharose beads (Roche). Total cell lysates and beads were separated using SDS-PAGE and probed with either anti-HA (Covance) or anti-phospho ERK antibodies (Cell Signaling).

### Yeast Two-Hybrid

YopJ wild type or C172A mutant cloned into the pLexAde vector (as bait) and Pbs2 or human MkK1 mutants cloned into the pVP16 vector (as prey) were transformed into yeast strain L40 using the same method mentioned above. Yeasts were grown on tryptophan- and leucine- deficient plates while two-hybrid interactions were tested using a colony-lift β-gal assay and visualized on nitrocellulose membranes [21].

## Supporting Information

Figure S1 (0.68 MB TIF)

Figure S2 (0.44 MB TIF)
