# Supplementary figures and images for: Structural Requirements for Yersinia YopJ Inhibition of MAP Kinase Pathways
Source: PLoS One. 2008 Jan 2;3(1):e1375. doi: 10.1371/journal.pone.0001375 (PMC2147050; doi:10.1371/journal.pone.0001375)

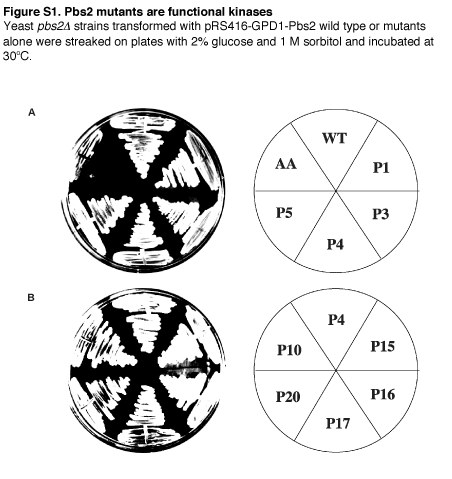

Supplement: Figure S1 — (0.68 MB TIF) [file pone.0001375.s001.tif]

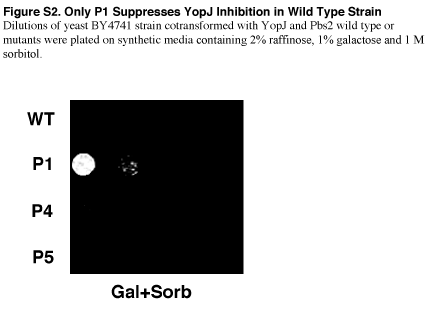

Supplement: Figure S2 — (0.44 MB TIF) [file pone.0001375.s002.tif]
